# Next-Generation Sequencing Refines Diagnosis and Expands Precision Medicine Opportunities in Soft Tissue Sarcomas

**DOI:** 10.3390/ijms27167201

**Published:** 2026-08-12

**Authors:** Francine Tesser-Gamba, Thais Biude Mendes, Fernanda Teresa Lima, Simone de Campos Vieira Abib, Eliana Maria Monteiro Caran, Silvia Regina Caminada de Toledo

**Affiliations:** 1 Genetics Laboratory, Pediatric Oncology Institute (IOP/GRAACC), Federal University of Sao Paulo, Sao Paulo 04039-001, SP, Brazil; 2 National Science and Technology Institute for Children’s Cancer Biology and Pediatric Oncology—INCT BioOncoPed, Porto Alegre 90010-150, RS, Brazil; 3 Department of Gynecology, Federal University of Sao Paulo, Sao Paulo 04024-002, SP, Brazil; 4 Department of Pediatric Surgery, Pediatric Oncology Institute (IOP/GRAACC), Federal University of Sao Paulo, Sao Paulo 04023-062, SP, Brazil; simoneabib@uol.com.br; 5 Department of Pediatrics Pediatric Oncology Institute (IOP/GRAACC), Federal University of Sao Paulo, Sao Paulo 04023-062, SP, Brazil

**Keywords:** pediatric soft tissue sarcoma, molecular profiling, next-generation sequencing, gene fusion, sarcoma diagnostics, precision oncology, targeted therapy

## Abstract

Soft tissue sarcomas (STSs) are a heterogeneous group of rare mesenchymal malignancies with overlapping morphological and immunohistochemical features, often making definitive diagnosis challenging. Recent advances in next-generation sequencing (NGS) have enabled the identification of recurrent molecular alterations that contribute to tumor classification, prognostic stratification, and precision oncology approaches. This retrospective study aimed to evaluate the diagnostic and clinical impact of molecular profiling in pediatric soft tissue sarcomas using the Oncomine Childhood Cancer Research Assay (OCCRA) panel. Fifty-five frozen tumor samples representing 24 distinct soft tissue sarcoma subtypes were obtained from the Pediatric Oncology Institute -IOP/GRAACC/UNIFESP Biobank (B-053). Molecular analysis was performed using NGS to identify gene fusions, single nucleotide variants (SNVs), copy number variations (CNVs), and insertions/deletions (InDels). Clinically relevant molecular alterations were identified in 70% (37/55) of cases, including 18 fusion transcripts, 13 SNVs, 8 CNVs, and 6 InDels. Recurrent and diagnostically relevant alterations included *BCOR*::*CCNB3*, *ASPSCR1*::*TFE3*, *NFR1:*:*BRAF*, *FUS*::*DDIT3*, *EML4*::*NTRK3*, *ETV6*::*NTRK3*, *CIC*::*DUX4*, *NAB2*::*STAT6* and *SS18*::*SSX1/2* fusions, as well as amplifications involving *PDGFRA*, *FGFR1*, *GLI1*, *CDK4*, *ERBB3*, and *KIT*. Pathogenic variants affecting genes involved in tumor suppression and chromatin remodeling, including *TP53*, *NF1*, *DICER1*, *SMARCA4*, *PTEN*, and *PIK3CA*, were also detected. Importantly, molecular profiling had significant diagnostic impact in several histologically ambiguous tumors, enabling molecular reclassification and refinement of previously inconclusive or inaccurate pathological diagnoses. In multiple cases, NGS transformed descriptive histopathological interpretations into genetically defined sarcoma entities, including *NTRK*-rearranged spindle cell neoplasms, *CIC*-rearranged sarcomas, synovial sarcoma, low-grade fibromyxoid sarcoma, and clear cell sarcoma. Furthermore, the identification of actionable alterations highlighted potential opportunities for targeted therapies and precision medicine approaches. Our findings demonstrate that comprehensive molecular profiling significantly enhances diagnostic accuracy in pediatric soft tissue sarcomas, particularly in morphologically challenging cases. The integration of NGS into routine sarcoma diagnostics enables biologically informed tumor classification and supports personalized therapeutic strategies.

## 1. Introduction

Soft tissue sarcomas (STSs) comprise a rare and highly heterogeneous group of mesenchymal malignancies encompassing more than 100 histological subtypes, each characterized by distinct biological behavior, clinical outcomes, and molecular drivers [1,2]. Although individually uncommon, these tumors represent a major diagnostic challenge in pediatric and young adult oncology due to their remarkable morphological diversity and overlapping histopathological features [1,3]. In many cases, conventional histopathological evaluation and immunohistochemistry are insufficient to establish a definitive diagnosis, particularly in poorly differentiated, spindle cell, round cell, or myxoid neoplasms [3,4]. Consequently, even experienced pathologists may encounter significant difficulty distinguishing between histologically similar entities, leading to inconclusive diagnoses or potential diagnostic misclassification [1,2,3,4].

Over the last decade, advances in molecular pathology have profoundly transformed the classification and diagnostic approach of soft tissue sarcomas [5,6,7,8]. The identification of recurrent genomic alterations, including fusion transcripts, copy number alterations, gene amplifications, and pathogenic mutations, has progressively shifted sarcoma classification from a morphology-based system toward an integrated molecular framework [1,5]. As highlighted in recent updates of the World Health Organization (WHO) Classification of Soft Tissue and Bone Tumors, many sarcoma entities are now defined primarily by their molecular alterations rather than exclusively by histological appearance [1,6].

Recent studies have emphasized that molecular diagnostics are becoming indispensable in contemporary sarcoma pathology, particularly in diagnostically challenging tumors [5,6,7,8]. Comprehensive molecular profiling not only improves diagnostic precision but also refines tumor classification, resolves ambiguous cases, and identifies clinically actionable biomarkers with direct therapeutic implications [5,7,8].

Although NGS has been increasingly incorporated into routine oncologic practice, the diagnostic impact of comprehensive molecular profiling in pediatric soft tissue sarcomas remains incompletely characterized, particularly in real-world cohorts from developing countries, where access to advanced molecular diagnostics is still limited and clinicopathological data remain scarce [9,10,11]. Moreover, most currently available studies have primarily focused on the identification of molecular alterations or the broad clinical applicability of genomic profiling, whereas comparatively few have systematically investigated the role of NGS in diagnostic refinement, molecular reclassification, and resolution of histologically challenging sarcomas [10,11].

Accurate diagnosis of STS continues to represent a major challenge due to substantial morphological overlap among entities, limited specificity of immunophenotypic markers, and the expanding recognition of molecularly defined tumor subtypes incorporated into contemporary classification systems [6,12]. In recent years, NGS has emerged as a powerful and increasingly indispensable diagnostic modality capable of detecting recurrent gene fusions, copy number alterations, and pathogenic sequence variants that are frequently critical for definitive tumor classification and biologically informed subclassification [6,10,12]. Importantly, integrated molecular profiling may substantially modify diagnostic interpretation, prognostic stratification, and therapeutic decision-making, particularly in poorly differentiated, undifferentiated, or diagnostically ambiguous tumors, with direct implications for precision oncology strategies [3,11,13].

In this context, the present study aimed to characterize the molecular landscape of pediatric STS using the Oncomine Childhood Cancer Research Assay (OCCRA) panel and to evaluate the diagnostic and clinical impact of targeted NGS in a real-world cohort of challenging pediatric sarcomas. Our findings demonstrate that comprehensive molecular profiling not only identifies clinically relevant and potentially actionable genomic alterations but also significantly enhances diagnostic precision, enabling the transition from descriptive morphology-based diagnoses to genetically defined sarcoma entities with direct clinical, prognostic, and therapeutic relevance. Collectively, these findings support the integration of molecular diagnostics into routine pediatric sarcoma pathology workflows and reinforce the central role of genomic profiling in the era of precision medicine.

## 2. Results

### 2.1. Patient and Sample Characteristics

Table 1 shows the clinical characteristics of patients with STS included in this study. The median age at diagnosis was 11 years (range, 0–30 years), and 22 patients (40%) were male. Among the 55 patients, 16 presented with metastatic disease at diagnosis. During follow-up, 15 patients experienced disease recurrence; of these, eight were alive with no evidence of disease at the last follow-up, six died from disease progression, and one was lost to follow-up.

### 2.2. Genetic Variants in STS Tumor Samples

Molecular profiling had substantial diagnostic impact in a significant subset of cases. Among the 23 tumors harboring gene fusions, 9 cases underwent diagnostic reclassification after NGS analysis due to the identification of disease-defining molecular alterations. In several tumors with ambiguous morphology, inconclusive immunohistochemistry, or poorly differentiated histology, the OCCRA panel enabled refinement from descriptive pathological diagnoses into genetically defined sarcoma entities. These findings directly influenced clinical interpretation, prognostic assessment, and potential therapeutic strategies.

**Figure 1:**
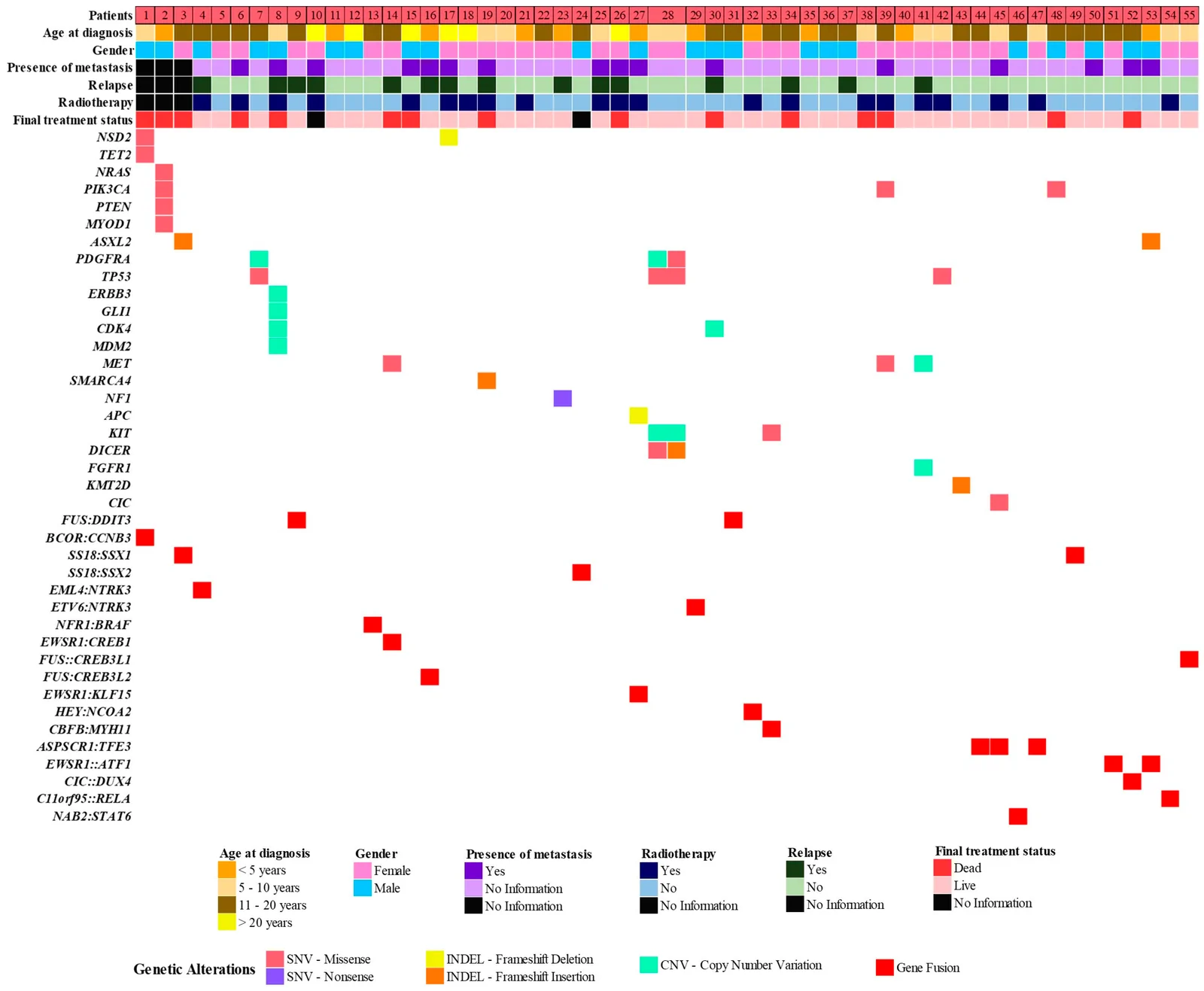
**Clinical and Molecular Landscape of Soft Tissue Sarcomas**—Each column represents one patient, and each row represents a clinical characteristic or molecular alteration. Colors indicate the different categories of clinical variables and molecular alterations identified.

NGS analysis identified a broad spectrum of genomic alterations, including missense mutations, nonsense mutations, frameshift deletions, insertions, copy number variations (CNVs), and gene fusions (Figure 1) The genes included in the targeted NGS panel and their corresponding HUGO Gene Nomenclature Committee (HGNC) identifiers are provided in Supplementary Table S1.

Gene fusions represented one of the most significant findings in the cohort and included recurrent rearrangements such as *BCOR::CCNB3*, *SS18::SSX1*/2, *EWSR1*::*CREB1*, *FUS*::*CREB3L2*, *EWSR1*::*KLF15*, *HEY1*::*NCOA2*, *ASPSCR1*::*TFE3*, *CIC*::*DUX4*, *NAB2*::*STAT6*, *ETV6*::*NTRK3*, and *EML4*::*NTRK3*. Of the 55 patients studied, 23 presented gene fusion. Of these 23 patients, in 9 the OCCRA panel was essential for defining and changing the diagnosis (Table 2). The cases in which there was a change in diagnosis and clinical management are described below.

Patient #4—A spindle cell/myxoid sarcoma initially diagnosed by histopathology was reclassified after NGS identified an *EML4::NTRK3* fusion, a defining alteration of infantile fibrosarcoma (IFS). This finding established the diagnosis of *NTRK* fusion-positive IFS and revealed potential eligibility for TRK inhibitor therapy, emphasizing the diagnostic and therapeutic relevance of molecular profiling in pediatric sarcomas [14,15].

Patient #13—A tumor initially classified as chondrosarcoma demonstrated an *NRF1::BRAF* fusion on molecular analysis. This rearrangement is exceptionally rare in mesenchymal neoplasms and has been primarily described in transitional cell carcinoma (TCC)/urothelial carcinoma, where activation of the MAPK signaling pathway may confer sensitivity to MEK-targeted therapies. The identification of this fusion suggests an unusual molecular subtype with potential therapeutic relevance and highlights the importance of comprehensive genomic profiling in diagnostically challenging sarcomas [16,17].

Patient #16—A fibroblastic sarcoma diagnosed by histopathology was reclassified as low-grade fibromyxoid sarcoma (LGFMS) following identification of a *FUS::CREB3L2* fusion by NGS. This recurrent and highly characteristic alteration confirmed the diagnosis and resolved the morphologic ambiguity commonly associated with LGFMS [18,19].

Patient #24—An undefined sarcoma was molecularly characterized by the identification of an *SS18::SSX2* fusion, establishing the diagnosis of synovial sarcoma. This canonical rearrangement enabled definitive classification of a morphologically indeterminate lesion and reinforced the role of molecular diagnostics in soft tissue sarcoma classification [1,20].

Patient #27—Histopathological findings initially supported extraskeletal myxoid chondrosarcoma (EMC); however, molecular analysis identified an *EWSR1::KLF15* fusion instead of the characteristic *NR4A3*-related rearrangements typically observed in EMC. These findings favored reclassification as a myoepithelial neoplasm with myxoid features, highlighting the diagnostic complexity of myxoid soft tissue tumors and the importance of genomic profiling [21,22].

Patient #51—A low-grade myxoid mesenchymal neoplasm of uncertain histogenesis demonstrated an *EWSR1*::*ATF1* fusion on OCCRA panel analysis. This rearrangement is a defining molecular hallmark of clear cell sarcoma, supporting diagnostic reclassification despite the atypical morphology and indeterminate immunophenotype. The identification of this fusion was particularly relevant because the lesion did not fit any currently recognized WHO thyroid tumor entity, indicating that molecular profiling was essential to establish the underlying sarcoma lineage. Given that clear cell sarcoma is an aggressive translocation-associated sarcoma with distinct biological and clinical behavior, the molecular findings had direct implications for tumor classification, prognosis, and therapeutic management [23,24].

Patient #52—A tumor initially diagnosed as Ewing sarcoma was reclassified after NGS identified a *CIC::DUX4* fusion, excluding conventional Ewing sarcoma defined by *EWSR1::ETS* rearrangements. This finding established the diagnosis of *CIC*-rearranged sarcoma, a biologically aggressive entity associated with poor prognosis and limited response to standard Ewing sarcoma chemotherapy [25,26].

Patient #53—A cutaneous lesion initially diagnosed as melanoma based on morphology and immunophenotype demonstrated an *EWSR1::ATF1* fusion on molecular analysis, supporting reclassification as clear cell sarcoma. This distinction is clinically significant due to major differences in tumor biology, prognosis, and therapeutic management between clear cell sarcoma and conventional melanoma [23,24].

Patient #55—A central nervous system (CNS) tumor initially interpreted as biphasic synovial sarcoma was reclassified following NGS identification of a *C11orf95::RELA* fusion, a defining molecular alteration of *ZFTA* fusion-positive ependymoma. The absence of the canonical *SS18::SSX* fusion further excluded synovial sarcoma. Considering the prominent sarcomatous morphology associated with the molecular profile of *RELA*-fused ependymoma, the findings support the diagnosis of ependymosarcoma, a rare variant of ependymoma characterized by mesenchymal/sarcomatous differentiation. This case highlights the essential role of molecular diagnostics in resolving challenging CNS tumor differentials and refining the classification of morphologically unusual ependymal neoplasms [27,28].

Additional pathogenic or potentially pathogenic alterations were detected in genes involved in cell-cycle regulation, receptor tyrosine kinase signaling, chromatin remodeling, and PI3K/AKT signaling pathways, including *TP53*, *PIK3CA*, *PTEN*, *NRAS*, *MET*, *KIT*, *PDGFRA*, *FGFR1*, *CDK4*, *MDM2*, *ERBB3*, *APC*, *NF1*, *SMARCA4*, *DICER1*, and *TET2*. Copy number alterations affecting oncogenic drivers such as *CDK4*, *MDM2*, *FGFR1*, and *KIT* were also identified (Figure 1). Detailed sequencing metrics and molecular variant annotations are provided in Supplementary Table S2.

### 2.3. Diagnostic Impact of OCCRA Panel Sequencing

Importantly, genomic profiling using the OCCRA panel had a major impact on diagnostic definition in a subset of patients. In 9 cases, the initial histopathological and immunohistochemical evaluation was insufficient to establish a definitive diagnosis due to overlapping morphological features and poorly differentiated tumor patterns (Table 2).

In these cases, molecular findings obtained through NGS were decisive for tumor classification and diagnostic refinement. Identification of specific gene fusions and characteristic molecular signatures enabled reclassification into distinct sarcoma entities, directly impacting the final diagnosis.

Notably, several diagnoses could only be conclusively established after detection of pathognomonic rearrangements, reinforcing the value of NGS as a complementary diagnostic tool in challenging pediatric soft tissue sarcomas. These findings demonstrate the clinical utility of the OCCRA panel in resolving diagnostically ambiguous cases that could not be accurately classified by conventional pathology alone.

### 2.4. Clinico-Molecular Correlations

Integrated analysis of clinical and molecular data demonstrated that patients with more complex genomic profiles—characterized by the coexistence of multiple somatic alterations, including single nucleotide variants (SNVs), insertions/deletions (indels), copy number variations (CNVs), and gene fusions—exhibited a more aggressive clinical course. These cases were frequently associated with metastatic disease at diagnosis, higher relapse rates, and unfavorable outcomes, including mortality, suggesting that increased mutational burden and genomic complexity may serve as indicators of poor prognosis.

Alterations affecting key oncogenic pathways, including TP53, PI3K/AKT signaling (*PIK3CA*/*PTEN*), MAPK pathway components (*NRAS* and *BRAF*-related fusions), and cell cycle regulators such as *CDK4* and *MDM2*, were enriched in molecular subgroups associated with more aggressive clinical behavior, supporting their biological relevance in tumor progression.

In addition, potentially actionable genomic alterations involving receptor tyrosine kinase signaling pathways were identified, including *NTRK3*, *FGFR1*, *KIT*, *PDGFRA*, and *MET*. These findings underscore the potential clinical utility of comprehensive molecular profiling, not only for refined diagnostic classification, but also for risk stratification and the identification of candidates for targeted therapeutic approaches within a precision oncology framework.

Collectively, these results indicate that molecular profiling provides clinically relevant complementary information to conventional clinical parameters, enabling the delineation of biologically and prognostically distinct tumor subsets and supporting more individualized therapeutic strategies.

## 3. Discussion

Soft tissue sarcomas (STSs) comprise a highly heterogeneous group of mesenchymal malignancies characterized by remarkable morphological diversity and complex molecular landscapes. Although histopathological examination and immunohistochemistry remain the cornerstone of diagnosis, many sarcomas present overlapping morphological features, nonspecific immunophenotypes, or poorly differentiated patterns that make definitive classification challenging, even for experienced pathologists [1]. In recent years, the increasing recognition of molecularly defined sarcoma entities has transformed the diagnostic approach to these tumors, establishing NGS as an essential tool in modern sarcoma pathology and an integral component of the current diagnostic workflow for soft tissue sarcomas [4,29,30,31].

While previous studies have demonstrated the clinical utility of NGS in sarcoma characterization, the impact of comprehensive molecular profiling on diagnostic refinement and molecular reclassification in histologically ambiguous soft tissue sarcomas remains underexplored [6,8,32,33]. In this study, we demonstrate that NGS not only confirms histopathological hypotheses but frequently redefines diagnostically challenging tumors into biologically and clinically actionable molecular entities.

These findings are consistent with the growing body of evidence supporting the integration of NGS-based molecular testing into the diagnostic workflow of soft tissue sarcomas. Recent international consensus recommendations advocate the use of NGS, particularly in diagnostically challenging cases, when morphology and immunophenotype are inconclusive, when confirmation of a characteristic gene fusion or genomic alteration is required for accurate tumor classification, or when molecular findings may influence prognostic assessment and therapeutic decision-making. Furthermore, real-world multicenter studies have demonstrated that comprehensive genomic profiling significantly improves diagnostic accuracy, refines tumor classification, and identifies clinically actionable alterations in routine clinical practice. Likewise, recent studies have reinforced the value of targeted RNA sequencing for the detection of recurrent and rare gene fusions that are critical for the diagnosis of soft tissue sarcomas. Our findings are in agreement with these observations and further support the implementation of targeted NGS panels as a practical and clinically applicable strategy for the molecular diagnosis of pediatric soft tissue sarcomas [30,31,34].

Using the OCCRA panel, clinically relevant molecular alterations were identified in 70% of cases, including fusion transcripts, single nucleotide variants (SNVs), copy number variations (CNVs), and insertions/deletions (InDels). Importantly, among tumors harboring gene fusions, molecular profiling led to diagnostic reclassification in multiple cases initially considered inconclusive or incorrectly classified based solely on morphology and immunohistochemistry.

Several cases highlighted the substantial diagnostic impact of molecular profiling. In one patient initially diagnosed with spindle cell/myxoid sarcoma, identification of an *EML4*::*NTRK3* fusion supported reclassification to *NTRK* fusion-positive infantile fibrosarcoma, a diagnosis with direct therapeutic implications due to the availability of TRK inhibitors [35,36]. Similarly, a tumor initially interpreted as fibroblastic sarcoma was reclassified as low-grade fibromyxoid sarcoma following detection of the highly characteristic *FUS*::*CREB3L2* fusion, considered a molecular hallmark of this entity [37].

In another diagnostically challenging case, molecular detection of *SS18*::*SSX2* established the diagnosis of synovial sarcoma in a tumor previously classified as undefined sarcoma. The *SS18*::*SSX* fusion is a defining molecular alteration of synovial sarcoma and plays a central role in tumor pathogenesis [20,38]. Likewise, identification of the *CIC*::*DUX4* fusion excluded conventional Ewing sarcoma and supported reclassification as *CIC*-rearranged sarcoma, a biologically distinct and clinically aggressive entity associated with poorer prognosis and reduced responsiveness to conventional Ewing sarcoma-based chemotherapy [25,39].

NGS also proved critical in distinguishing tumors with overlapping morphologic and immunophenotypic features. A lesion initially diagnosed as melanoma based on expression of melanocytic markers was reclassified as clear cell sarcoma after identification of the disease-defining *EWSR1*::*ATF1* fusion. This distinction is clinically significant, as clear cell sarcoma differs substantially from melanoma regarding biological behavior, metastatic patterns, and therapeutic management [24,40].

Additionally, molecular profiling enabled recognition of rare or emerging molecular entities, including *BCOR*::*CCNB3* sarcoma, *NTRK*-rearranged spindle cell neoplasms, and tumors harboring *EWSR1*-related fusions. These findings reinforce the evolving concept that many sarcomas are now best classified according to their molecular drivers rather than exclusively by morphology [1,4,36,41,42].

High-grade soft tissue sarcomas (STS) represent a biologically diverse group of mesenchymal malignancies characterized by complex genomic alterations, frequent chromosomal instability, and heterogeneous clinical outcomes. Despite histologic classification, increasing evidence supports that integrated molecular profiling—incorporating copy number variations (CNVs), single nucleotide variants (SNVs), and gene fusions—provides superior biologic stratification and may better predict clinical behavior than morphology alone [18,29,40]. The present cohort highlights recurrent alterations affecting key oncogenic pathways, including cell cycle regulation, receptor tyrosine kinase signaling, and epigenetic modulation, many of which correlate with aggressive clinical courses.

A striking observation in this series was the strong association between *CDK4* amplification and adverse outcome, as both patients harboring *CDK4* CNVs developed metastases, local recurrence, and ultimately died of disease. *CDK4* is a central regulator of the G1–S cell cycle checkpoint, and its amplification has been widely reported in aggressive sarcomas, particularly well-differentiated and dedifferentiated liposarcomas, but also in undifferentiated pleomorphic sarcomas and other high-grade STS. Functional activation of the *CDK4/6–RB* axis leads to unchecked proliferation and resistance to senescence, and clinical studies have consistently demonstrated that *CDK4* amplification correlates with poor prognosis and reduced response to conventional therapies. These findings support *CDK4* not only as a prognostic biomarker but also as a potential therapeutic target in selected sarcoma subsets [43,44,45].

Similarly, amplification events involving *MDM2*, *ERBB3*, and *GLI1* in one patient with aggressive clinical behavior underscore the convergence of oncogenic signaling pathways in high-grade sarcomas. *MDM2* amplification functionally inactivates *TP53*, promoting genomic instability and tumor progression. *ERBB3* activation is increasingly recognized as a driver of PI3K/AKT signaling in solid tumors, while *GLI1* activation reflects aberrant Hedgehog pathway signaling, which has been implicated in sarcomagenesis and tumor stemness. The co-occurrence of these CNVs likely reflects a highly unstable genomic landscape associated with treatment resistance and metastatic potential [46,47,48,49,50,51,52,53].

Another case demonstrated concurrent CNVs involving *MET* and *DICER1*, followed by tumor recurrence. *MET* amplification leads to constitutive activation of the hepatocyte growth factor receptor pathway, promoting proliferation, invasion, and angiogenesis. *MET*-driven sarcomas have been associated with aggressive clinical behavior and resistance to chemotherapy. *DICER1* alterations, although classically linked to syndromic tumor predisposition, have been increasingly described in sporadic mesenchymal tumors and are thought to disrupt microRNA processing, contributing to widespread transcriptomic dysregulation [54,55]. The coexistence of these events suggests synergistic activation of proliferative signaling and loss of post-transcriptional control, favoring tumor progression.

The identification of *PIK3CA* and *PTEN* alterations, often within the same or across multiple cases, highlights the central role of the PI3K/AKT/mTOR pathway in sarcoma biology [56,57]. Activating mutations in *PIK3CA* combined with *PTEN* loss result in constitutive pathway activation, promoting survival and growth advantage. Importantly, these alterations were observed across different histologic subtypes, including high-grade sarcoma, synovial sarcoma, and spindle cell sarcoma, supporting the concept that *PI3K* pathway activation represents a convergent oncogenic mechanism independent of lineage differentiation. This finding is consistent with prior genomic studies demonstrating frequent activation of *PI3K* signaling across diverse sarcoma subtypes and provides a rationale for targeted therapeutic approaches.

Of particular interest, recurrent PIK3CA pathogenic SNVs were identified across three different patients with high-grade sarcoma, synovial sarcoma, and spindle cell sarcoma. This reinforces the role of PI3K pathway activation as a recurrent and potentially targetable event across morphologically distinct sarcomas. The clinical relevance of this pathway has been increasingly supported by early-phase clinical trials evaluating PI3K and mTOR inhibitors in advanced sarcoma cohorts [58].

The presence of pathogenic *TP53* mutations in three patients with distinct histologies (spindle cell sarcoma, clear cell sarcoma, and embryonal sarcoma) further underscores the role of tumor suppressor loss in sarcomagenesis. Although *TP53* alterations are generally associated with genomic instability and poor outcome, the present cases showed variable clinical courses, suggesting that *TP53* mutation alone is insufficient to define prognosis and must be interpreted in the context of co-occurring genomic alterations and tumor biology. This aligns with recent studies demonstrating that *TP53*-mutant sarcomas display marked molecular heterogeneity, chromosomal instability, and divergent evolutionary trajectories associated with aggressive clinical behavior [29,53].

Additionally, *ASXL2* mutations were identified in synovial sarcoma and clear cell sarcoma cases. *ASXL2* is an epigenetic regulator involved in chromatin remodeling and transcriptional control. Although its role in sarcoma is not fully established, alterations in *ASXL* family genes have been implicated in epigenetic dysregulation across multiple malignancies [59]. In the synovial sarcoma case, *ASXL2* mutation co-occurred with the canonical *SS18*::*SSX1* fusion, a defining molecular hallmark of synovial sarcoma. The coexistence of epigenetic disruption and fusion-driven oncogenesis suggests additional layers of transcriptional deregulation that may contribute to tumor aggressiveness and phenotypic plasticity.

In case #2, the histopathological diagnosis of a high-grade sarcoma with rhabdomyosarcomatous differentiation is supported by the molecular profile, which revealed a pathogenic *MYOD1* variant in association with additional alterations in *NRAS***,** *PIK3CA*, and *PTEN*. This combination raises the strong possibility that the lesion represents a *MYOD1*-mutant spindle/sclerosing rhabdomyosarcoma, a distinct and well-recognized high-risk entity within the spectrum of rhabdomyosarcomas. The presence of *MYOD1* mutation is particularly significant, as it is strongly associated with aggressive clinical behavior, rapid tumor progression, and a poor prognosis compared with other rhabdomyosarcoma subtypes. Furthermore, concurrent dysregulation of the MAPK (*NRAS*) and PI3K/AKT/mTOR (*PIK3CA* and *PTEN*) signaling pathways suggests a convergent oncogenic mechanism driving sustained proliferation and survival, which may contribute to treatment resistance [58]. In this context, the initial diagnostic question of whether the lesion represents a true rhabdomyosarcoma is effectively resolved in favor of a molecularly defined rhabdomyosarcoma entity, with the genetic findings providing strong confirmatory and prognostic support for the diagnosis and indicating a high-risk disease category that warrants intensive multimodal therapy and consideration of clinical trial enrollment.

Overall, the present cohort illustrates that high-grade sarcomas frequently harbor overlapping oncogenic alterations affecting core biological pathways, including cell cycle regulation (*CDK4*, *MDM2*), receptor tyrosine kinase signaling (*MET*, *ERBB3*), PI3K/AKT/mTOR signaling (*PIK3CA*, *PTEN*), developmental transcription factors (*MYOD1*), and epigenetic regulators (*ASXL2*). Importantly, cases with *CDK4* amplification demonstrated uniformly poor outcomes, highlighting its potential role as a robust adverse prognostic biomarker in this setting. These findings reinforce the importance of integrated molecular diagnostics in sarcoma pathology. Beyond histologic classification, genomic profiling enables the identification of biologically meaningful subgroups with distinct clinical behavior and potential therapeutic vulnerabilities. As targeted therapies continue to evolve, particularly CDK4/6 inhibitors, MET inhibitors, and PI3K pathway inhibitors, molecular stratification will likely become increasingly relevant for guiding precision oncology approaches in soft tissue sarcomas [45,54,58,60]. Based on these findings, we can state that NGS transformed descriptive histopathological diagnoses into biologically defined molecular entities.

Beyond its diagnostic value, comprehensive molecular profiling identified several genomic alterations with potential therapeutic implications. Although most of these therapies are not currently considered standard of care for pediatric soft tissue sarcomas, identification of these alterations may support treatment selection in relapsed or refractory disease and facilitate enrollment in biomarker-driven clinical trials. These findings reinforce the role of comprehensive NGS not only in diagnostic refinement but also in precision oncology by identifying molecular vulnerabilities that may inform future therapeutic strategies.

Among the clinically actionable findings, the *EML4*::*NTRK3* fusion identified in one patient represents a well-established predictive biomarker for TRK inhibitors such as larotrectinib and entrectinib, both of which have demonstrated remarkable and durable responses across NTRK fusion-positive solid tumors, including pediatric sarcomas. Likewise, recurrent alterations involving the PI3K/AKT/mTOR pathway (*PIK3CA* mutations and *PTEN* loss) represent potential candidates for *PI3K*, *AKT*, or *mTOR* inhibitors currently under clinical investigation in sarcoma. *CDK4* amplification, observed in two patients with aggressive clinical behavior, provides a biological rationale for CDK4/6 inhibitors, while *MET* amplification represents a potentially targetable alteration with MET inhibitors. Although these targeted approaches are not currently standard of care for most pediatric soft tissue sarcomas, their identification may support enrollment in molecularly matched clinical trials and expand future therapeutic opportunities [35,36,61,62].

Importantly, our findings underscore the limitations of conventional histopathology when used in isolation. In several cases, descriptive pathological diagnoses such as “undifferentiated sarcoma,” “spindle cell neoplasm,” or “myxoid mesenchymal tumor” were transformed into genetically defined entities after NGS analysis. This integrated diagnostic approach significantly improves diagnostic accuracy, refines prognostic stratification, and directly impacts clinical decision-making.

Despite these advantages, some limitations of targeted NGS panels should be acknowledged. Because the OCCRA is restricted to predefined genes and fusion partners, genomic alterations outside its targeted content—including rare, novel, or yet uncharacterized events—may not be detected. This limitation is particularly relevant in soft tissue sarcomas, whose molecular classification continues to expand with the discovery of new genetic drivers. In diagnostically unresolved cases or tumors lacking canonical molecular alterations, broader genomic approaches such as whole-transcriptome RNA sequencing, whole-exome sequencing, or whole-genome sequencing may provide additional diagnostic information. Nevertheless, as demonstrated in the present cohort, the OCCRA panel encompasses the majority of clinically relevant alterations currently incorporated into the diagnosis and management of pediatric soft tissue sarcomas, providing a rapid, cost-effective, and clinically applicable strategy for routine molecular diagnostics.

This study highlights the substantial diagnostic impact of comprehensive genomic profiling, particularly in cases with inconclusive histopathological findings. The ability of the OCCRA panel to redefine or establish diagnoses in 9 patients underscores its practical relevance in real-world clinical oncology. The cohort revealed a broad and heterogeneous molecular spectrum, including rare fusion events and clinically relevant alterations across multiple sarcoma subtypes. Finally, the integration of genomic and clinical data provided important insights into associations between molecular alterations, metastatic behavior, relapse, and survival outcomes.

Overall, these findings support the incorporation of comprehensive NGS profiling into routine diagnostic workflows for pediatric and young adult sarcomas, particularly in diagnostically challenging tumors, while also expanding the current understanding of the molecular landscape of these rare malignancies.

Importantly, although most patients in this retrospective cohort were treated according to standard therapeutic protocols, identification of actionable molecular alterations provided clinically meaningful information for future therapeutic decision-making. In recurrent or refractory disease, these genomic findings could support personalized treatment strategies or inclusion in biomarker-driven clinical trials, illustrating that comprehensive NGS contributes not only to diagnostic accuracy but also to precision oncology.

In conclusion, comprehensive molecular profiling by NGS significantly enhances diagnostic precision in soft tissue sarcomas, particularly in morphologically ambiguous or histologically inconclusive tumors. The integration of molecular diagnostics into routine sarcoma evaluation allows the transition from descriptive histopathological interpretation to biologically defined molecular classification, with important prognostic and therapeutic implications. Our findings further underscore the critical role of integrated histopathological assessment and comprehensive molecular profiling as a fundamental framework for precision medicine in soft tissue sarcoma pathology, enabling more accurate diagnostic classification, refined risk stratification, and improved identification of therapeutically actionable alterations.

## 4. Materials and Methods

### 4.1. Patients and Samples

A total of 55 fresh-frozen STS samples were obtained from 55 patients treated at the Pediatric Oncology Institute (IOP/GRAACC–UNIFESP) (Table 3). Tumor specimens were prospectively collected during surgical procedures and immediately cryopreserved in the institutional pediatric oncology biobank. At the time of collection, all specimens were reviewed by an experienced pathologist to ensure representative viable tumor tissue, maximizing tumor cellularity while minimizing the inclusion of ne-crotic and non-neoplastic areas. Tumor cellularity was estimated by histopathological evaluation of hematoxylin and eosin (H&E)-stained sections, considering the proportion of viable tumor cells relative to the total nucleated cells in the selected tissue. All samples included in this study presented an estimated tumor cell content greater than 80%. All samples belong to the Institute’s Biobank (CONEP–B-053) and were stored at −80 °C until extraction. This study was approved by the Research Ethics Committee of the Federal University of São Paulo (CEP/UNIFESP No. 0715P/2021), and written informed consent was obtained from all patients or their legal guardians.

### 4.2. DNA and RNA Extraction and Quantification

Total DNA and RNA were extracted from frozen tumor samples using FastPrep^®^-24 Tissue Ruptor (MP Biomedicals^®^, Santa Ana, CA, USA) and AllPrep DNA/RNA Mini Kit (QIAGEN^®^, Hilden, Germany) according to the manufacturer’s instructions. After extraction, samples were quantified in Nanodrop 2000^®^ and in Qubit 3^®^ fluorometer using Qubit RNA HS Assay Kit^®^ and Qubit dsDNA HS Assay Kit^®^ (Thermo Fisher Scientific^®^, Waltham, MA, USA). The ratio between the 260 nm and 280 nm absorbances was used as quality control (QC) for the extractions. An A260/280 ratio between 1.6 and 1.8 and 1.8–2.0 was adopted for DNA and RNA, respectively. Only samples with the expected QC standards were included in the study. After quantification, synthesis of complementary DNA (cDNA) was performed using 20 ng of total RNA according to the manufacturer’s protocol of SuperScript Vilo IV^®^ (Thermo Fisher Scientific^®^).

### 4.3. Library Preparation and Next-Generation Sequencing (NGS) Run with OCCRA© Panel

Sequencing libraries were prepared from 20 ng of DNA (15 μL) and 20 ng of cDNA (13 μL) from each tumor sample using the Ion Chef™ System (Thermo Fisher Scientific) according to the manufacturer’s instructions. Libraries were loaded onto Ion 540™ Chips and sequenced on the Ion S5™ System using the Oncomine™ Childhood Cancer Research Assay (OCCRA) panel, following the manufacturer’s protocol [63,64].

### 4.4. QC Acceptability Criteria

Quality control (QC) was performed before and after sequencing. Samples were accepted only when homopolymer regions contained fewer than eight consecutive bases, library concentration met assay requirements, and sequencing quality scores were ≥Q20. Copy number analysis was considered reliable only for samples with a Median Absolute Pairwise Difference (MAPD) ≤0.35. Variants identified in low-quality regions were considered potential false positives [63,64].

### 4.5. Oncomine Childhood Cancer Research Assay (OCCRA©) Sequencing Panel Analysis

Sequence alignment and variant calling were performed using Ion Reporter™ version: 5.20 software with the human reference genome GRCh37 (hg19). Variants were annotated according to the Human Genome Variation Society (HGVS) recommendations using the corresponding RefSeq transcript (NM accession), and detailed variant information is provided in Supplementary Table S2.

## Figures and Tables

**Table 1:** Clinical characteristics of soft tissue sarcoma patients.

		N (%)
**Age at diagnosis**	<5	11 (20)
	5–10	13 (24)
	11–20	25 (45)
	>20	6 (11)
**Gender**	Female	33 (60)
	Male	22 (40)
**Primary location**	Thigh	12 (22)
	Arm	7 (13)
	Femur	6 (11)
	Pelvis/Sacrum/Pelvis	4 (7)
	Liver	4 (7)
	Brain	5 (9)
	Tibia	3 (5)
	Cervical/thoracic	2 (4)
	Armpit	2 (4)
	Toedactyl	1 (2)
	Abdomen	1 (2)
	Face	1 (2)
	Inguinal	1 (2)
	Tongue	1 (2)
	Gluteus	1 (2)
	Orbit	1 (2)
	Kidney	1 (2)
	Foot	1 (2)
	Thyroid	1 (2)
**Metastasis**	Yes	16 (29)
	No	36 (66)
	No information	3 (5)
**Radiotherapy**	Yes	21 (39)
	No	31 (56)
	No information	3 (5)
**Relapse**	Yes	15 (27)
	No	37 (68)
	No information	3 (5)
**Final Status**	Alive	37 (68)
	Dead	15 (27)
	No information	3 (5)

**Table 2:** Diagnostic Refinement Based on OCCRA Molecular Findings in Selected Sarcoma Cases.

# Patient	Diagnostic Hypothesis (Pathological Anatomy)	OCCRA Panel Molecular Finding	Diagnostic Definition
4	Spindle cell/myxoid sarcoma	*EML4::NTRK3*	Infantile fibrosarcoma
13	Chondrosarcoma/Reactive Lymphoid Hyperplasia	*NRF1::BRAF*	Transitional Cell Carcinoma
16	Fibroblastic Sarcoma	*FUS::CREB3L2*	Low-Grade Fibromyxoid Sarcoma
24	Soft tissue sarcoma	*SS18::SSX2*	Sarcoma Sinovial
27	Extraskeletal myxoid chondrosarcoma	*EWSR1::KLF15*	Myoepithelial Carcinoma of Soft Tissue
51	Thyroid tumor with invasion of the trachea and recurrent laryngeal nerve on the right	*EWSR1::ATF1*	Clear cell sarcoma
52	Ewing’s sarcoma with lung metastasis at diagnosis	*CIC::DUX4*	High-grade undifferentiated round cells sarcoma
53	Melanoma	*EWSR1::ATF1*	Clear cell sarcoma
54	Biphasic Synovial Sarcoma	*C11orf95::RELA*	Ependymosarcoma

**Table 3:** Histopathological diagnoses of the patients included in the study.

Patient	Diagnosis
1	High-grade undifferentiated sarcoma
2	High-grade sarcoma/Rhabdomyosarcoma
3	Synovial Sarcoma
4	Spindle cell/myxoid sarcoma
5	Neurofibrosarcoma
6	Leiomyosarcoma
7	High-grade spindle cell and epithelioid sarcoma
8	High-grade malignant sarcoma
9	Myxoid liposarcoma
10	Myxoid liposarcoma
11	Spindle cell sarcoma with myxoid areas
12	Chondrosarcoma
13	Chondrosarcoma
14	Clear cell sarcoma
15	Angiosarcoma
16	Fibroblastic sarcoma
17	Mesenchymal Chondrosarcoma
18	Myxoid chondrosarcoma
19	Chondrosarcoma
20	High-grade spindle cell sarcoma/fibrosarcoma
21	Schwannoma V pair/Intraorbital Fibromyxoid Sarcoma
22	High-grade spindle cell sarcoma
23	Hepatic Sarcoma
24	Soft tissue sarcoma
25	High-grade spindle cell sarcoma
26	Leiomyosarcoma
27	Extraskeletal myxoid chondrosarcoma
28	Clear Cell Sarcoma
29	Childhood fibrosarcoma
30	High-grade pleomorphic sarcoma
31	Myxoid liposarcoma
32	Mesenchymal chondrosarcoma
33	Granulocytic sarcoma
34	Spindle cell pattern sarcoma
35	Non-rhabdomyosarcoma sarcoma with myxoid pattern
36	Low-grade chondrosarcoma
37	Low-grade epithelioid and spindle cell pattern sarcoma
38	Hepatosarcoma
39	Synovial Sarcoma—High-grade spindle cell sarcoma
40	Childhood fibrosarcoma
41	High-grade sarcoma (spindle cell and epithelioid)
42	High-grade hepatic embryonal sarcoma
43	Embryonal sarcoma
44	Alveolar sarcoma
45	Alveolar soft tissue sarcoma
46	Fibromyxoid sarcoma with low proliferative index
47	Alveolar sarcoma
48	Spindle cell pattern sarcoma
49	Synovial sarcoma of the left knee
50	Alveolar soft tissue sarcoma
51	Clear cell sarcoma
52	High-grade undifferentiated round cells sarcoma
53	Clear cell sarcoma
54	Ependymosarcoma
55	Low-grade fibromyxoid sarcoma

## Data Availability

All data generated or analyzed during this study are included in the article and its Supplementary Materials. Further inquiries can be directed to the corresponding author.

## Supplementary Materials

The following supporting information can be downloaded at: https://www.mdpi.com/article/10.3390/ijms27167201/s1.
