# Supplementary material for: Next-Generation Sequencing Refines Diagnosis and Expands Precision Medicine Opportunities in Soft Tissue Sarcomas
Source: Int J Mol Sci. 2026 Aug 12;27(16):7201. doi: 10.3390/ijms27167201 (PMC13513196; doi:10.3390/ijms27167201)
Supplement: Supplementary file 1 [file ijms-27-07201-s001.zip › Supplementary table S1.pdf]

Supplementary table S1 - Full names corresponding to all gene symbols used in the manuscript.

Gene symbol	HGNC ID	Official full gene name
APC	HGNC:583	APC regulator of WNT signaling pathway
ASPSCR1	HGNC:13825	ASPSCR1 tether for SLC2A4, UBX domain containing
ASXL2	HGNC:23805	Additional sex combs like transcriptional regulator 2
ATF1	HGNC:783	Activating transcription factor 1
BRAF	HGNC:1097	B-Raf proto-oncogene, serine/threonine kinase
BCOR	HGNC:20893	BCL6 corepressor
CCNB3	HGNC:18709	Cyclin B3
CDK4	HGNC:1773	Cyclin dependent kinase 4
CIC	HGNC:14214	Capicua transcriptional repressor
C11orf95	HGNC:28449	Chromosome 11 open reading frame 95 (historical symbol; currently renamed ZFTA)
CREB1	HGNC:2345	cAMP responsive element binding protein 1
CREB3L2	HGNC:23720	cAMP responsive element binding protein 3 like 2
DICER1	HGNC:17098	Dicer 1, ribonuclease III
DUX4	HGNC:50800	Double homeobox 4
EML4	HGNC:1316	Echinoderm microtubule associated protein like 4
ERBB3	HGNC:3431	Erb-b2 receptor tyrosine kinase 3
ETS	HGNC:3488	ETS transcription factor family (gene family, not a single gene)
ETV6	HGNC:3495	ETS variant transcription factor 6
EWSR1	HGNC:3508	EWS RNA binding protein 1
FGFR1	HGNC:3688	Fibroblast growth factor receptor 1
FUS	HGNC:4010	FUS RNA binding protein
GLI1	HGNC:4317	GLI family zinc finger 1
HEY1	HGNC:4880	Hes related family bHLH transcription factor with YRPW motif 1
KIT	HGNC:6342	KIT proto-oncogene, receptor tyrosine kinase
KLF15	HGNC:14536	Kruppel like factor 15
MDM2	HGNC:6973	MDM2 proto-oncogene
MET	HGNC:7029	MET proto-oncogene, receptor tyrosine kinase
MYOD1	HGNC:7611	Myogenic differentiation 1
NAB2	HGNC:7627	NGFI-A binding protein 2
NCOA2	HGNC:7669	Nuclear receptor coactivator 2
NF1	HGNC:7765	Neurofibromin 1
NR4A3	HGNC:7982	Nuclear receptor subfamily 4 group A member 3
NRAS	HGNC:7989	NRAS proto-oncogene, GTPase
NRF1	HGNC:7996	Nuclear respiratory factor 1
NTRK3	HGNC:8033	Neurotrophic receptor tyrosine kinase 3
PDGFRA	HGNC:8803	Platelet derived growth factor receptor alpha
PIK3CA	HGNC:8975	Phosphatidylinositol-4,5-bisphosphate 3-kinase catalytic subunit alpha
PTEN	HGNC:9588	Phosphatase and tensin homolog
RELA	HGNC:9955	RELA proto-oncogene, NF-kB subunit
SMARCA4	HGNC:11100	SWI/SNF related, matrix associated, actin dependent regulator of chromatin, subfamily A, member 4
SS18	HGNC:11340	SS18 subunit of BAF chromatin remodeling complex
SSX1	HGNC:11335	SSX family member 1
SSX2	HGNC:11336	SSX family member 2
STAT6	HGNC:11368	Signal transducer and activator of transcription 6
TET2	HGNC:25941	Tet methylcytosine dioxygenase 2
TFE3	HGNC:11752	Transcription factor binding to IGHM enhancer 3
TP53	HGNC:11998	Tumor protein p53
TRK	HGNC:8031	Tropomyosin receptor kinase
ZFTA	HGNC:28449	Zinc finger translocation associated

HGNC - HUGO Gene Nomenclature Committee
