# Supplementary material for: Next-Generation Sequencing Refines Diagnosis and Expands Precision Medicine Opportunities in Soft Tissue Sarcomas
Source: Int J Mol Sci. 2026 Aug 12;27(16):7201. doi: 10.3390/ijms27167201 (PMC13513196; doi:10.3390/ijms27167201)
Supplement: Supplementary file 1 [file ijms-27-07201-s001.zip › Supplementary table S2.pdf]

Supplementary table S2 - Sample quality, Sequencing metrics and Variant annotation.

	Sample quality sequencing metrics				Variantion annotation								
Sample ID	Mapped Reads	On-target (%)	Mean depth (×)	Uniformity (%)	Gene / Gene fusion	Type	HGVS	Fusion nomenclature (VICC)	dbSNP*	Clinical significance	Coverage (variant)	VAF
1	10,264,397	98.34	3.985	97.91	NSD2	SNV ; INDEL	NM_001042424.3:c.4022C>A p.(Pro1341His) ; NM_001042424.3:c.4028del p.(Pro1343GlnfsTer49)	N/A	rs774265779 ; rs752037034	Uncertain Significance ; Likely Pathogenic / Pathogenic	1970	49,5% / 29,5%
					TET2 / TET2-AS1	SNV	NM_001127208.3:c.5152G>T p.(Val1718Leu)	N/A	rs142312318	Benign / Likely Benign	1996	48,10%
					BCOR::CCNB3	fusion	N/A	BCOR::CCNB3, inv(X)(p11.4;p11.22), exon 15::exon 5	N/A	N/A	146947	N/A
2	10,226,815	98.35	3.987	98.08	NRAS	SNV	NM_002524.5:c.181C>A p.(Gln61Lys)	N/A	rs121913254	Conflicting Classifications Of Pathogenicity	1999	30%
					PIK3CA	SNV	NM_006218.4:c.3140A>G p.(His1047Arg)	N/A	rs121913279	Pathogenic	1999	18,40%
					PTEN	SNV	NM_000314.8:c.376G>T p.(Ala126Ser)	N/A	N/A	Uncertain Significance	1997	8,60%
					MYOD1	SNV	NM_002478.5:c.365T>G p.(Leu122Arg)	N/A	N/A	Likely Pathogenic / Pathogenic	1999	29%
3	10,197,624	98.27	3.977	97.26	ASXL2	INDEL	NM_018263.6:c.2332_2333insG p.(Pro778ArgfsTer26)	N/A	N/A	N/A	1964	51,70%
					SS18::SSX1	fusion	N/A	SS18::SSX1, t(X;18)(p11;q11), SS18 exon10::SSX1 exon6	N/A	N/A	78312	N/A
4	10,441,718	97.53	3.998	97.68	EML4::NTRK3	fusion	N/A	EML4::NTRK3, t(2;15)(p21;q25), EML4 exon2::NTRK3 exon14	N/A	N/A	189226	N/A
5	10,582,674	98.03	4.109	97.96	***	***	***	***	***	***	***	***
6	10,862,838	97.88	4.227	98.11	***	***	***	***	***	***	***	***
7	11,625,704	97.52	4.071	96.84	PDGFRA	CNV	N/A	N/A	N/A	N/A	6.26	N/A
					TP53	SNV	NM_000546.6:c.524G>A p.(Arg175His)	N/A	rs28934578	Pathogenic	1999	47,50%
8	10,502	97.71	4.054	98.16	ERBB3	CNV	N/A	N/A	N/A	N/A	8.14	N/A
					GLI1	CNV	N/A	N/A	N/A	N/A	8.54	N/A
					CDK4	CNV	N/A	N/A	N/A	N/A	12.35	N/A
					MDM2	CNV	N/A	N/A	N/A	N/A	21.55	N/A
9	11,466,825	97.55	4.44	98.15	FUS::DDIT3	fusion	**N/A**	FUS::DDIT3, t(12;16)(q13;p11), FUS exon5::DDIT3 exon2;3	N/A	N/A	8590 ; 30824	N/A
10	10,741,563	98.16	4.178	97.96	***	***	***	***	***	***	***	***
11	10,186,044	99.05	4.028	96.26	***	***	***	***	***	***	***	***
12	10,124,441	98.21	3.953	98.23	***	***	***	***	***	***	***	***
13	10,674,917	97.77	4.13	97.98	NRF1::BRAF	fusion	N/A	NRF1::BRAF, t(7;7)(q32;q34), NRF1 exon5::BRAF exon9	N/A	N/A	53570	N/A
14	10,179,895	97.85	3.959	98.22	MET	SNV	NM_001127500.3:c.3029C>T p.(Thr1010Ile)	N/A	rs56391007	Conflicting Classifications Of Pathogenicity	1998	49,40%
					EWSR1::CREB1	fusion	N/A	EWSR1::CREB1, t(2;22)(q33;q12), EWSR1 exon7::CREB1 exon7	N/A	N/A	86078	N/A
15	10,452,373	97.79	4.062	98.26	***	***	***	***	***	***	***	***
16	10,578,519	98.26	4.113	98.14	FUS::CREB3L2	fusion	N/A	FUS::CREB3L2, t(7;16), FUS exon6::CREB3L2 exon5	N/A	N/A	65370	N/A
17	10,869,089	98.08	4.186	97.42	NSD2	INDEL;SNV	NM_001042424.3):c.4028del p.(Pro1343GlnfsTer49) ; NM_001042424.3):c.4023C>T p.(Pro1341=)	N/A	rs752037034 ; rs149925711	Likely Pathogenic / Pathogenic ; Benign Likely Benign	1936	36,8% / 54,8%
18	11,236,462	98.01	4.36	98.00	***	***	***	***	***	***	***	***
19	9,287,480	97.77	3.638	97.90	SMARCA4	INDEL	NM_001128849.3:c.995_996insT p.(Gly334ArgfsTer53)	N/A	rs1013942482	Uncertain Significance	1967	43,60%
20	9,064,338	97.64	3.52	97.97	***	***	***	***	***	***	***	***
21	9,566,973	97.64	3.704	97.99	***	***	***	***	***	***	***	***
22	9,049,551	97.43	3.488	97.87	***	***	***	***	***	***	***	***
23	12,476,402	98.34	4.858	98.06	NF1	SNV	NM_001042492.3:c.2041C>T p.(Arg681Ter)	N/A	rs768638173	Pathogenic	1999	97,40%
24	10,038,301	98.12	3.883	97.96	SS18::SSX2	fusion	N/A	SS18::SSX2, t(X;18)(p11.2;q11.2), SS18 exon10::SSX2 exon6	N/A	N/A	30078	N/A
25	11,689,176	98.09	4.514	97.94	***	***	***	***	***	***	***	***
26	12,062,719	97.92	4.687	97.96	***	***	***	***	***	***	***	***
27	12,467,759	97.84	4.82	98.19	APC	INDEL	NM_000038.6:c.3920del p.(Ile1307LysfsTer14)	N/A	rs1064794229	Pathogenic	1990	52,70%
					EWSR1::KLF15	fusion	N/A	EWSR1::KLF15, t(3;22)(q21;q12), EWSR1 exon7;8::KLF15 exon2	N/A	N/A	2388 ; 76703	N/A
28	10,922,921	97.78	4.18	97.04	PDGFRA	CNV	N/A	N/A	N/A	N/A	6.7	N/A
					PDGFRA	SNV	NM_006206.6:c.1682T>A p.(Val561Asp)	N/A	rs121908586	Conflicting Classifications Of Pathogenicity	1999	77%
					KIT	CNV	N/A	N/A	N/A	N/A	5.65	N/A
					DICER1	SNV	NM_030621.4:c.5125G>A p.(Asp1709Asn)	N/A	rs1595331264	Pathogenic	2000	49,30%
					DICER1	INDEL	NM_030621.4:c.4331dup p.(Tyr1445ValfsTer2)	N/A	N/A	Uncertain Significance	1994	46%
					TP53	SNV	NM_000546.6:c.659A>G p.(Tyr220Cys)	N/A	rs121912666	Pathogenic	1657	86%
29	10,119,871	98.02	4.005	97.86	ETV6::NTRK3	fusion	N/A	ETV6::NTRK3, t(12;15)(p13;q25), ETV6 exon5::NTRK3 exon15	N/A	N/A	8645	N/A
30	9,321,864	98.20	3.649	98.14	CDK4	CNV	N/A	N/A	N/A	N/A	5.95	N/A
31	10,518,171	97.74	4.141	97.72	FUS::DDIT3	fusion	N/A	FUS::DDIT3, t(12;16)(q13;p11), FUS exon5::DDIT3 exon2;3	N/A	N/A	9245 / 25227	N/A
32	10,730,010	97.52	4.181	97.81	HEY1::NCOA2	fusion	N/A	HEY1::NCOA2, t(8;8)(q13;q21), HEY1 exon4::NCOA2 exon13	N/A	N/A	192057	N/A
33	10,554,104	98.17	4.143	97.36	KIT	SNV	NM_000222.3:c.2446G>T p.(Asp816Tyr)	N/A	rs121913506	Uncertain Significance	1996	44,30%
					CBFB::MYH11	fusion	N/A	CBFB::MYH11, t(16;16)(p13;q22), CBFB exon5::MYH11 exon33	N/A	N/A	87263	N/A
34	9,932,531	97.45	3.867	97.79	***	***	***	***	***	***	***	***
35	11,337,557	97.96	4.439	97.50	***	***	***	***	***	***	***	***
36	10,891,828	97.68	4.288	97.08	***	***	***	***	***	***	***	***
37	9,932,263	97.76	3.836	97.57	***	***	***	***	***	***	***	***
38	10,373,084	97.96	4.032	97.86	***	***	***	***	***	***	***	***
39	10,127,068	98.28	3.946	98.23	PIK3CA	SNV	NM_006218.4:c.1035T>A p.(Asn345Lys)	N/A	rs121913284	Pathogenic	1996	21,60%
					MET	SNV	NM_001127500.3:c.3029C>T p.(Thr1010Ile)	N/A	rs56391007	Conflicting Classifications Of Pathogenicity	1998	50%
40	9,699,944	98.04	3.731	98.17	***	***	***	***	***	***	***	***
41	10,229,032	97.41	3.916	98.10	MET	CNV	N/A	N/A	N/A	N/A	13.55	N/A
					FGFR1	CNV	N/A	N/A	N/A	N/A	11.79	N/A
42	9,419,493	97.16	3.583	98.06	TP53	SNV	NM_000546.6:c.817C>T p.(Arg273Cys)	N/A	rs121913343	Likely Pathogenic / Pathogenic	1999	7,40%
43	9,051,313	97.72	3.47	97.84	KMT2D	INDEL	NM_003482.4:c.2992_2993insA p.(Pro998HisfsTer70)	N/A	N/A	N/A	1943	51,20%
44	11,460,095	98.25	4.53	98.13	ASPSCR1::TFE3	fusion	N/A	ASPSCR1::TFE3, t(X;17)(p11;q25), ASPSCR1 exon7::TFE3 exon5	N/A	N/A	78282	N/A
45	10,260,915	97.69	4.002	97.67	ASPSCR1::TFE3	fusion	N/A	ASPSCR1::TFE3, t(X;17)(p11;q25), ASPSCR1 exon7::TFE3 exon5	N/A	n/A	58850	***
					CIC	SNV,MNV	NM_015125.5:c.4533_4534delCCinsTT p.(Arg1512Cys) ; NM_015125.5):c.4533C>T p.(Ile1511=)	N/A	N/A ; rs1052023	Uncertain Significance ; Benign	1997	97% / 2,9%
46	10,416,970	97.84	4.056	97.97	NAB2::STAT6	fusion	N/A	NAB2::STAT6, inv12(q13q13), NAB2 exon6::STAT6 exon16;17	N/A	N/A	201917 / 18806	N/A
47	9,008,696	97.70	3.532	97.62	ASPSCR1::TFE3	fusion	N/A	ASPSCR1::TFE3, t(X;17)(p11;q25), ASPSCR1 exon7::TFE3 exon6	N/A	N/A	70396	N/A
48	9,151,538	98.26	3.525	97.21	PIK3CA	SNV	NM_006218.4:c.1624G>A p.(Glu542Lys)	N/A	rs121913273	Pathogenic	1996	30%
49	9,389,722	97.78	3.6	98.27	SS18::SSX1	fusion	N/A	SS18::SSX1, t(X;18)(p11;q11), SS18 exon10::SSX1 exon6	N/A	N/A	57677	N/A
50	9,891,934	98.13	3.846	98.27	***	***	***	***	***	***	***	***
51	9,991,264	97.81	3.855	98.13	EWSR1::ATF1	fusion	N/A	EWSR1::ATF1, t(12;22)(q13;q12), EWSR1 exon7::ATF1 exon4	N/A	N/A	6537	N/A
52	11,873,580	97.85	4.667	96.53	CCND3	SNV	NM_001760.5:c.851C>T p.(Pro284Leu)	N/A	rs2127389288	Uncertain Significance	2000	3,50%
					SMARCA4	SNV	NM_001128849.3:c.2292G>A p.(Trp764Ter)	N/A	rs2088999811	Pathogenic	2000	3,20%
					CIC::DUX4	fusion	N/A	CIC::DUX4, t(4;19)(q35;q13), CIC exon20::DUX4 exon1	N/A	N/A	44418	N/A
53	11,743,837	97.78	4.522	98.10	ASXL2	INDEL	NM_018263.6:c.2332_2333insG p.(Pro778ArgfsTer26)	N/A	rs1271466699	Uncertain Significance	1953	52%
					EWSR1::ATF1	fusion	N/A	EWSR1::ATF1, t(12;22)(q13;q12), EWSR1 exon7::ATF1 exon5	NA	N/A	133659	N/A
54	7,478,433	98.28	2.934	97.57	C11orf95::RELA	fusion	N/A	C11orf95::RELA, 11q13.1, C11orf95 exon3::RELA exon2	NA	N/A	10888	N/A
55	10,132,983	98.99	3.983	97.21	FUS::CREB3L1	fusion	N/A	FUS::CREB3L1, t(11;16)(p11;p11), FUS exon6::CREB3L1 exon5	NA	N/A	317236	N/A

*** No variants were detected in the sample using the OCCRA panel.
N/A Not Applicable
VICC - Gene Fusion Specification
* dbSNP identifiers are provided when available; variants without an assigned rs identifier are indicated as N/A.
